# Shaping Eyeballs by Scleral Collagen Cross-Linking: A Hypothesis for Myopia Treatment

**DOI:** 10.3389/fmed.2021.655822

**Published:** 2021-07-02

**Authors:** Mengmeng Wang, Christine Carole C. Corpuz, Fengju Zhang

**Affiliations:** ^1^Hebei Ophthalmology Key Lab, Hebei Eye Hospital, Xingtai, China; ^2^Ifugao State University Eye Center, Alfonso Lista, Philippines; ^3^Beijing Tongren Eye Center, Beijing Tongren Hospital, Capital Medical University, Beijing, China

**Keywords:** myopia, sclera remodeling, scleral collagen cross-linking, sub-tenon's injection, staphyloma

## Abstract

The global prevalence of myopia has brought to the attention of the different eye and vision specialists, who make way to control its progression. Evidence have shown that a proactive reshaping of the eyeball is the core point of myopia developing process, which particularly includes the weakening, thinning, and expanding of the sclera. Thus, the sclera is considered to be a prime target for therapeutic manipulation in halting progressive myopia. In the past decades, corneal collagen cross-linking has been applied in clinical practice for treating aberrant corneal remodeling diseases. In this article, we hypothesize that scleral collagen cross-linking (SXL) has a huge potential in stabilizing myopic process by shaping the eyeball and preventing the aberrant scleral remodeling. In contrast with the current methods of optometry correction, such as physiotherapy, pharmacotherapy, spectacles, contact lenses, refractive surgeries, etc., eyeball-shaping method using SXL is a fundamental intervention which aims at the pathogenesis of progressive visual loss of myopia. Compared with the current posterior scleral reinforcement, the most advantage of SXL is that there is no allotransplant into the myopic eye, which means less expenditure, lower risk, and easier to handle in operating.

## Introduction

Myopia is the most common cause of visual impairment worldwide (1). It was speculated that the global prevalence of myopia by 2050 will be about 4,758 million (49.8%), including 277 million (4.0% of the global population) of high myopia (2). The burden of myopia is tremendous, as adults with high myopia are more likely to develop pathologic myopia (PM) changes, such as posterior scleral staphyloma, choroidal neovascularization, retinal choroidal atrophy, streak, peripheral retinal degeneration, retinal detachment, and finally lead to blindness (3–5). Generally, myopia was believed to be a mismatch of the eye's anatomical axial length and its focal length (6) which was caused by a “multifactorial” mechanism (7, 8). Aberrant scleral remodeling is the core point during the myopic process, and has been the goal of any long-term therapy for the persistent vision loss associated with myopia (9). Collagen cross-linking, introduced by Wollensak et al. (10), is an effective approach to increase the biomechanical strength of the corneal and scleral tissue. Corneal collagen cross-linking (CXL) has been applied in clinical practice for treating aberrant corneal remodeling diseases in the past decades (11). Similarly, scleral collagen cross-linking (SXL) has a huge potential in stabilizing myopic process by shaping the eyeball and preventing the aberrant scleral remodeling, such as ocular axial elongation and staphyloma.

## Hypothesis

We hypothesize SXL as an eyeball-shaping method to prevent the aberrant scleral remodeling during myopic process. Such a theory has not been conceptualized yet, and has not been verified by clinical trials. We reason that the strengthening the integral sclera by SXL could slow down the process of diffuse expansion during eyeball growth; strengthening the equatorial sclera by SXL could limit the axial elongation of the progressive myopic eyeball; partially strengthening the sclera by SXL could block the local bulges which causes staphyloma. Due to its cross-linking effect only on the outer layer of the sclera, SXL is a safe method for the function of the inner tissues underneath the sclera. SXL could be artificially carried out by different cross-linking categories, doses, frequencies, sites according to the current status and prognosis of myopic eyes. For these reasons, it will prevent or halt the progression of myopia in any stage of the disease.

## Aberrant Scleral Remodeling of Myopia

There are strong evidences from clinical and experimental studies indicating that the biochemical and biomechanical properties of the sclera play a major role in the progression of myopia (12). Thinning of the sclera and weakened biomechanical properties, particularly at the posterior pole of the eye, have long been known to be an important feature in the development of high myopia in human and mammalian models (9). It is found that myopic sclera undergoes a proactive reshaping process which includes diameter decrease and fiber gap enlargement of the collagenous fiber bundle in all parts of the myopic eye, particularly in the posterior sclera; this causes retinal fundus complications which adversely affect visual acuity (9, 12). Thus, the sclera is considered to be a prime target for therapeutic manipulation in halting progressive myopia (13). Currently, scleral reinforcement is the only available clinical option to slow down scleral bulges (such as in staphyloma), which remain controversial due to its complication risk (14). Thus, given the lack of treatment options, novel clinical solutions are becoming increasingly necessary available to halt progressive scleral remodeling and morbidity of progressive myopia.

## Principle of SXL

Cross-linking is a natural phenomenon in the body following an enzymatic or a non-enzymatic pattern (15). Generally, the phenomenon increases in age (16) or in diabetics (17), who rarely show progressive myopia (18). On the contrary, diminished cross-linking is an important factor in the weakening process of myopic sclera (16) with a significant decrease in Young's modulus (19). It is therefore hypothesized that, instead of natural cross-linking, artificially-induced cross-linking might be used therapeutically to retard axial elongation, thereby reducing the risk of blindness.

Photochemically-induced crosslinkings, which currently include riboflavin/UV light crosslinking and riboflavin/blue light crosslinking, can create additional chemical bonds between collagen fibers by photopolymerization and increase scleral stiffness (20). This photochemically-induced cross-linking is mediated by photooxidation between riboflavin (vitamin B2) and laser light (UV light, 360–370 nm; blue light, 400–500 nm). The laser light activates riboflavin into triplet, which in turn produces reactive oxygen species (ROS) and singlet oxygen. ROS reacts with collagen fibril molecules in the corneal stroma and enhances the mechanical strength of cornea by forming new chemical bonds between amino groups of collagen fibril molecules (21). Theoretically, blue light has better penetration ability and lower damage in sclera compared to UV light considering the negative relation between wavelength and penetrating depth in tissue (22).

Chemically-induced crosslinking is another kind of artificially-induced cross-linking, which is usually applied by injecting of chemical crosslinking agents into the sub-Tenon's (sT) space and scleral surface. This kind of crosslinking could represent a simpler way to treat the posterior sclera, thus, avoiding the need for UV light exposure (23). Although there are many compounds that are suitable for industrial and commercial cross-linking purposes, only a limited number of compounds have emerged with potential for scleral stiffening *in vivo*: glyceraldehyde (24), genipin (25–27), methylglyoxal (28, 29) (also called pyruvaldehyde), formaldehyde releasers (FARs) (30), sodium hydroxymethylglycinate (SMG) (31), methylglyoxal (32). Applying the concept of the Maillard reaction (15), these crosslinking agents can be added to the ends of protein molecules and can be further transformed to advanced glycation end products, which are more stable. The resultant covalent collagen cross-links promote enhanced tissue stiffness and resistance to enzymatic degradation (33).

## Technique of SXL

Until now, no protocol of photochemically-induced crosslinking was set up for sclera treatment, while several protocols of corneal collagen cross-linking has been suggested to be used on corneal stroma for the treatment of keratoconus, such as the Dresden protocol (34, 35), Siena University protocol (36), Athens protocol (37). Based on the experimental parameters reported in previous studies, the operating procedure of photochemically-induced SXL was generally as follows: after adequate exposure of the target scleral surface, a UV/blue light-source was set up to irradiate directly on the target scleral surface. Specific intensity and energy were performed to ensure that the exposure of UV/blue light on the cornea is below harmful levels. A syringe with a blunt needle allowed an almost continuous supply of the riboflavin photosensitizer solution during the irradiation (38).

Compared with the photochemical procedure, which requires a surgical procedure to access the posterior sclera with a UV light source, injecting into the sub-Tenon's space with chemical cross-linking agents is much simpler (27, 39). Like photochemically-induced crosslinking, no standardized protocol of chemically-induced crosslinking has been set up at present, especially in the parameters of drug concentration and dose (32). However, in contrast to photochemically-induced crosslinking, repeated sub-Tenon's injections of crosslinking agents can be performed in different quadrants for several times in the following sequential days, months, or years. Another advantage of chemically-induced crosslinking is that, with the help of ultrasound, the chemical crosslinking agents can easily be injected via sub-Tenon's approach, to the posterior globe and even the whole sclera, and could create much larger crosslinked areas than photochemically-induced crosslinking (33, 40).

## Known Facts About SXL

Photochemically-induced crosslinking has been proven to increase the mechanical strength of scleral collagen in guinea pigs (40), rabbits (41), pigs (10), primates (42), and human sclerae (43). A significant increase in the density and area of collagen fibrils and a significant decrease in the density and area of the interfibrillar spacing can be found in both the equatorial and posterior sclera with either riboflavin/UVA or riboflavin/blue light SXL for 12 months postoperatively (42–44). However, the UV/blue light irradiation with high intensities induces degenerative alterations of scleral cells mainly, in the episcleral and outer scleral layers, which is associated with extracellular matrix degradation, tissue inflammation, hemorrhage, and macrophage infiltration; and in the inner scleral layers, where treatment with high-intensity irradiation induces scleral cell activation with pronounced metabolic activity, and finally results in scleral scarring (45). Not only would the high light intensities be harmful, but also, complicated operations would bring dangerous side-effects on the ocular tissues of the crosslinked eye (46). For example, during the irradiation process, the eyes had to be stretched to expose the target scleral region. Irradiation was largely focused on the equatorial area, thereby inducing possible mechanical injury to ocular tissues. This approach made it impossible to fully expose the posterior sclera that is prone to develop staphyloma (47). Moreover, many other adverse factors were encountered in the past, such as complexity of operating riboflavin/UVA sclera collagen cross-linking, large lesions, difficulty in irradiating the posterior sclera, and difficulty in administering repetitive irradiation (48). Although a minimally invasive therapy has reported to be performed by inserting miniaturelightemitting diodes (LEDs) under the tenon's capsule to the posterior sclera without stretching the eyeball tissues (38), its drawbacks have been put forward, including limited irradiance, risk of thermal tissue damage, and poor flexibility (49).

One potential treatment modality to overcome the above adverse factors of SXL is chemically-induced crosslinking by sub-Tenon's injection of chemical crosslinking agents. This method could serve as a simple and minimally invasive approach to reach the posterior sclera without the need for any specialized UV device or UV irradiation (50). Our research team (39) has successfully used sub-Tenon's injection of 0.5% (22.1 mM) genipin to decelerate form-deprivation myopia in guinea pigs. Another previous study (33) also found that sub-Tenon's injection of 0.15 ml 0.5 M glyceraldehyde seven times during 14 days reduced the AXL elongation of rabbit eyes, with no retinal and choroidal affects under light microscopy. It was also found that glyceraldehyde could increase scleral stiffness for at least 8 months in rabbits (24). Compared with glyceraldehyde (seven fold) or methylglyoxal (30 fold), a much lower concentration of genipin is required to achieve a similar scleral stiffening effect after the sub-Tenon's injection, which means that SXL using genipin could be an alternative low-cytotoxic collagen crosslinking agents (32). Moreover, sub-Tenon's injection of FARs (30) and SMG (31) have also shown their effects in halting the progression of scleral elongation seen in myopic eyes, and would be potential cross-linking compounds. Nevertheless, it should be mentioned that the safety of chemically-induced crosslinking was not fully evaluated at present. In a previous study (51), a sub-conjunctival injection with glyceraldehyde resulted in intraocular pressure elevation and loss of retinal ganglion cell axon, which means that this type of SXL induced glaucoma in these eyes.

## Discussions and Predictions

As scleral crosslinking remains untested on human eyes, we believe that through continuous research and development, SXL would eventually become a novel method for shaping eyeballs and retarding myopic progression. In contrast with the current methods of optometry correction (spectacle works, contact lenses, refractive surgeries, etc.), eyeball-shaping method using SXL would be a fundamental intervention which aim at the pathogenesis of progressive visual loss of myopia. SXL will be also used for preventing and treating the myopia-related complications, such as staphyloma, tractive retinal detachment and macular cleave. Compared with the current posterior scleral reinforcement (PSR) (52), the most advantage of SXL is that there is no allotransplant into the myopic eye, which means less expenditure, lower risk, and easier to handle in operating.

For photochemically-induced crosslinking, several necessary developments on light delivery system will make this SXL method much safer than the current ones. The novel light delivery will provide homogeneous light distribution on scleral spherical surface. This light-delivery system can be easily and flexibly introduced and operated deep in the eye socket to deliver light to the relatively large target region with sufficient irradiance, short procedural time and reduced invasiveness. For accuracy, this system can provide a series of irradiation area size, from several square millimeters to hundred square millimeters, without any extra light leakage to the adjacent tissues. For safety, this system can create its SXL effects on the outer and medium layers of sclera, and can be no harmful for the deeper ocular tissues.

For chemically-induced crosslinking, outstanding crosslinking agents will be selected according to their efficacy under physiologic pH and temperature, permeability, and cell toxicity (53). Mature treatment strategies will be set up and used including the drug compositions, dose, injection sites, and repetition frequency based on the illness condition. Novel drug delivery systems, such as microneedles (54), sub-tenon (episcleral) implants (55), and transscleral iontophoresis (56), will be combined for increasing the bioavailability of crosslinking agents, improving their penetrability, reducing their systemic absorption and side effects, reducing administration frequency, and improving the comfort and compliance of patients.

Until now, the current SXL protocol has a lot of urgent questions to be answered. For example, dose-effect relationship and dose-toxicity relationship should be generalized. For photochemically-induced crosslinking, the light delivery systems should be initially optimized for less damage. For chemically-induced crosslinking, both effective and safe chemical crosslinking agents should be established using more comparative studies. Long-term observation of SXL efficacy should also be lasting for several years. Thus, there is still a long road ahead before clinical studies are conducted.

Because of their respective characteristics, both photochemically-induced crosslinking and chemically-induced crosslinking will be performed according to the myopic types and the treatment plan. Photochemically-induced crosslinking, which can be easily controlled in dosage, duration and area size of irradiation, will be applied on the symmetrical locations of sclera for axial myopia or on the partial sclera for staphyloma. For larger and homogeneous crosslinking effect, chemically-induced crosslinking can be repeatedly applied by multi-site injections around the equatorial sclera or even the whole sclera. Moreover, it should be noticed that SXL can only stabilize the situation of myopia but not improve vision. This means that SXL can only be useful on eyes while myopia is progressing but not when myopia has stabilized. Thus, we believe that a complex series of treatment, which includes not only both kinds of SXLs but also adding refractive corrections (spectacle works, contact lenses, refractive surgeries, etc.) and myopia-related complication treatments (photodynamic therapy, laser photocoagulation, anti-VEGF intravitreal injections, pars plana vitrectomy, etc.) will be applied in clinics according to the realistic conditions and treatment needs in the future.

## Data Availability Statement

The original contributions presented in the study are included in the article/supplementary material, further inquiries can be directed to the corresponding author/s.

## Author Contributions

MW and FZ collected the data as well as drafted and revised the manuscript. CC revised the manuscript. MW conceptualized and designed the hypothesis as well as reviewed the manuscript. All authors approved the final manuscript as submitted and agreed to be accountable for all aspects of the work.

## Conflict of Interest

The authors declare that the research was conducted in the absence of any commercial or financial relationships that could be construed as a potential conflict of interest.
